# Gut microbiota influence tumor development and Alter interactions with the human immune system

**DOI:** 10.1186/s13046-021-01845-6

**Published:** 2021-01-25

**Authors:** Yanshan Ge, Xinhui Wang, Yali Guo, Junting Yan, Aliya Abuduwaili, Kasimujiang Aximujiang, Jie Yan, Minghua Wu

**Affiliations:** 1 Hunan Provincial Tumor Hospital and the Affiliated Tumor Hospital of Xiangya Medical School, Central South University, Changsha, 410013 Hunan China; 2 Basic School of Medicine, Central South University, Changsha, 410078 Hunan China; 3 The Key Laboratory of Carcinogenesis of the Chinese Ministry of Health, The Key Laboratory of Carcinogenesis and Cancer Invasion of the Chinese Ministry of Education, Cancer Research Institute, Central South University, Changsha, 410008 Hunan China; 4 Basic School of Medicine, Xinjiang Medical University, Urumqi, 830011 Xinjiang China; 5 Department of Forensic Science, School of Basic Medical Sciences, Central South University, Changsha, 410078 Hunan China

**Keywords:** Gut microbiota, Immunity, Metabolism, Gut-brain/liver/lung axis

## Abstract

Recent scientific advances have greatly enhanced our understanding of the complex link between the gut microbiome and cancer. Gut dysbiosis is an imbalance between commensal and pathogenic bacteria and the production of microbial antigens and metabolites. The immune system and the gut microbiome interact to maintain homeostasis of the gut, and alterations in the microbiome composition lead to immune dysregulation, promoting chronic inflammation and development of tumors. Gut microorganisms and their toxic metabolites may migrate to other parts of the body via the circulatory system, causing an imbalance in the physiological status of the host and secretion of various neuroactive molecules through the gut-brain axis, gut-hepatic axis, and gut-lung axis to affect inflammation and tumorigenesis in specific organs. Thus, gut microbiota can be used as a tumor marker and may provide new insights into the pathogenesis of malignant tumors.

## Background

Human intestines harbor approximately 3.8 × 10^13^ microorganisms that maintain the physiology and health of the host by influencing basic functions, such as metabolism, nutrition, immunomodulation, and pathogen resistance [1–5]. Recently, gut microbiota have been reported to play key roles in the regulation of several processes related to brain function and mental health [6]. At the same time, gut microbiota play an important role in host disease pathogenesis [7]. Drugs and particular diseases (autoimmune and chronic diseases) may cause intestinal microbial dysfunction [8]. Other factors, such as activation of inflammatory signaling, dietary changes, infection, and lack of nucleotide-binding oligomerization domain 2 (NOD2), can also lead to dysbiosis [9, 10]. Dysbiosis of the microbiome has differential effects on the abundance of certain gut microbiota. It may increase metabolic disorders and the abundance of inflammation-inducing bacteria, which can induce carcinogenesis [11–13]. Gut microbiota regulates cancer at the level of genetic instability, susceptibility to host immune response, progression, and response to therapy [14, 15].

Using animal models, researchers have gained insights into the mechanisms through which microbes trigger carcinogenesis [16–18]. *Escherichia coli* and *Bacteroides fragilis* have been shown to potentiate intestinal tumorigenesis in chronic inflammation [19]. Besides, specific microbes and microbial dysbiosis have been shown to induce and even promote carcinogenesis by releasing genotoxins that may damage host DNA [20, 21]. Recent research shows, host innate immune responses against the resident microbiome may lead to tumor growth [16]. Thus, the gut microbiome is an essential factor for consideration in the precise treatment of cancer and can be used as a biomarker for diagnosis and treatment purposes [22]. Moreover, the efficacy of cancer treatments has been shown to be reduced in antibiotic-treated and germ-free mice, suggesting that intact gut microbiota is necessary for optimal treatment response [23].

Regardless of health or disease status, gut microbiota affect metabolism, tissue development, inflammation, and immunity in the host [24]. Of course, the large amount of communication in the gut-organ axis cannot be separated from the involvement of the gut microbiota. It has been shown that there are bidirectional interactions within the brain-gut-microbiome axis, involving neural, endocrine, and inflammatory mechanisms [25, 26]. In addition, intestinal flora has been shown to affect liver immune function and bile acid metabolism through the gut-liver axis [27]. Similarly, lung inflammation originating in the gut has been reported in a study of the gut-lung axis [28]. In this review, we summarize how gut dysbiosis and immune dysregulation can lead to the induction and maintenance of tumors. Moreover, alterations in the microbiome may participate in immune modulation to promote cancer through metabolic pathways. We propose that gut microbiota dysbiosis affects cancer development through the gut-organ axis.

## Gut microbiota dysbiosis is associated with the occurrence and development of cancer

The human gut is populated by trillions of archaea, bacteria, eukaryotes, viruses, and microbes belonging to four major microbial phyla: Proteobacteria, Firmicutes, Bacteroides, and Actinobacteria; these account for 95% of the gut microbiome [29]. Notably, true oncomicrobes in the intestine account for a very small proportion of all microbial populations. Furthermore, components of the microbiota such as flagellin can alter this balance and promote chronic inflammation, promoting intestinal tumor development [30]. HPV can cause the overexpression of the E6 and E7 genes of the virus, thereby cooperating to make the host cell immortal [31, 32]. Cunningly, microorganisms and their metabolites may migrate to other parts of the body and contribute to tumor development [32]. Disruption of the intestinal barrier function may trigger inflammation and carcinogenesis [12, 32]. Impaired barrier function can cause bacteria to enter the intestinal epithelium, allowing toxins to be transmitted. Bacterial toxins, such as colistin that is produced by *Escherichia coli*, have been shown to potentiate colorectal cancer in azoxymethane-exposed mice [12]. The toxin produced by the enterotoxigenic *Bacteroides fragilis* is related to colorectal tumors [33, 34]. In fact, microbes also drive cancerous transformation by affecting genome stability, resistance to cell death, and proliferation. For example, soluble fiber microbial fermentation disorders can induce cholestatic liver cancer [35]. Gut microbiota dysbiosis modulates the responses of CD8^+^ T cells to influence colitis-associated tumorigenesis [18]. Peptide tyrosine tyrosine expression induced by gut microbiota dysbiosis has been linked to the development of pancreatic cancer [36, 37]. Dysbiosis of gut microbiota has been shown to lead to the progression of chronic inflammation and liver disease, thereby increasing the risk of hepatocellular carcinoma (HCC) [38]. Interventions that regulate intestinal flora and improve immune function may be new regimens for future cancer treatment [29, 30]. Specific microbial changes can cause flora imbalance through signaling pathways and promote related cancer progression. Representative cancers related to gut dysbiosis are listed in Table 1.

**Table 1:** Table 1

Cancers Associated	microbiota	signaling	reference
With Gut Dysbiosis			
Pancreatic cancer	Proteobacteria, Bacteroidetes, Firmicutes	TLR	[37]
Colorectal cancer	Bifidobacteria, Helicobacter, Bacteroides	TGF-β	[39–41]
Liver cancer	Fiber-Fermenting Bacteria Proteobacteria	TLR	[35, 42]
Lung cancer	Enterococcus,Streptococcus, Prevotella		[43, 44]
Gastrointestinal cancer	*H. pylori*	STAT3	[45–47]
Breast cancer	*Pseudomonas aeruginosa*, human papilloma virus	NF-κΒ	[48, 49]
Thyroid cancer	Neisseria,Streptococcus	STAT3	[50]
Bladder cancer	*Bacteroides fragilis* and Clostridium cluster I	IL-6	[51, 52]

## Gut microbiome and immune dysregulation in cancer

Intriguingly, the gut microbiome can inhibit infection by intestinal pathogens by occupying a niche, adjusting the niche environment, competing for nutrients, releasing bacteriocins, and regulating host immune defense. This process starts during the constitution of the microbiome at birth, affecting the maturation of the immune system, the development of tolerance and containment of the microbiome [53, 54]. In the mucosa, the T and B cells of immune system have the phenotype and function of specific locations affected by the microflora. These cells play a key role in maintaining immune homeostasis by inhibiting the response to harmless antigens and preserving the integrity of the intestinal mucosal barrier function [54].

Indeed, the host intestinal mucosal surface barrier allows microbial symbiosis [45, 55]. Gut microbiota are susceptible to continuous damage caused by the environment and must be repaired quickly to restore homeostasis. Disruption of the gut barrier results in confrontation between the microorganisms and the immune system, which may result in cancer and inflammatory diseases. The immune response in the developing tumor microenvironment, including the triggering of pro-inflammatory or immunosuppressive processes, can be further affected by microorganisms [46].

**Fig. 1:**
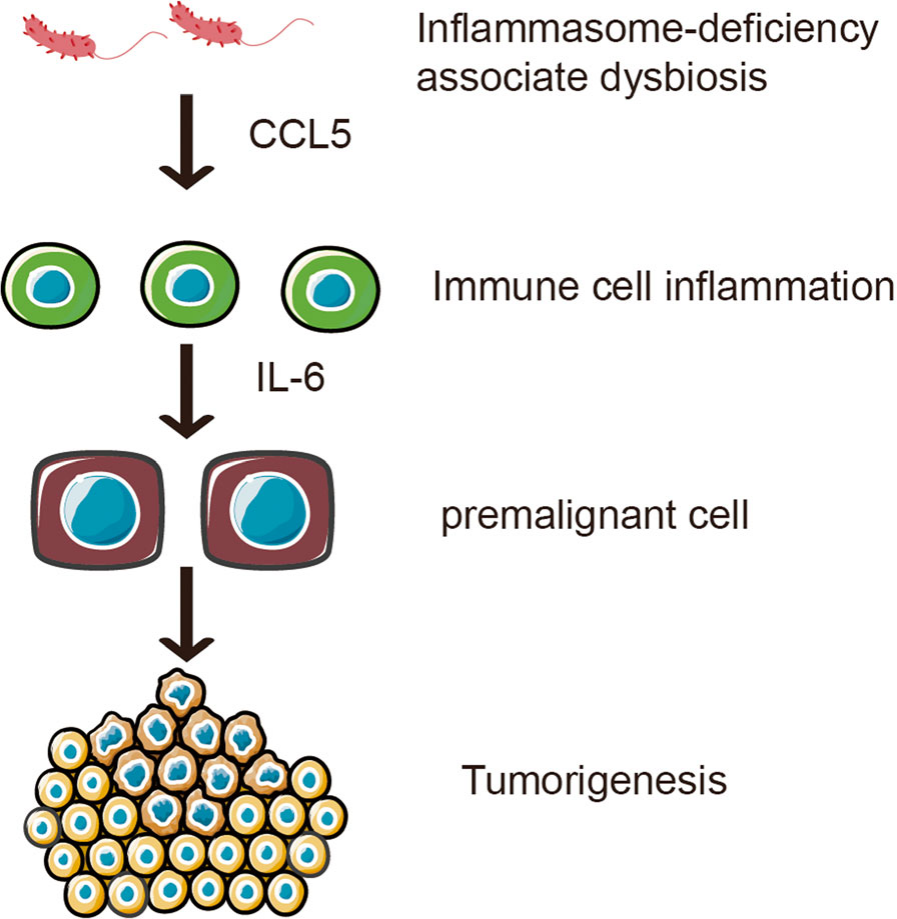
Gut dysbiosis can drive inflammation-induced cancer; it causes epithelial reprogramming and induces CCL5 transcription to induce local inflammation. In turn, it leads to local induction of IL-6 secretion and proliferation of intestinal epithelial cells, ultimately leading to tumor formation

The central role of immunity in the biology of cancer calls for attention to the exact contribution of microbiota in oncogenesis. For example, gut microbiome dysbiosis promotes inflammation via chemokine C-C chemokine ligand 5 (CCL5), which recruits a non-physiological number of lymphocytes in the intestine, and the resulting inflammatory state promotes epithelial cell proliferation through local activation of the interleukin-6 (IL-6) pathway [56, 57] (Fig. 1). Upregulation of toll-like receptors (TLRs) by lipopolysaccharide (LPS) and other microbial products can activate the nuclear factor (NF)-κB, c-Jun/JNK, and JAK/STAT3 pathways, which have well-defined roles in cell proliferation and immunosuppression [21, 50].

### Gut microbiome participates in immune modulation to promote cancer through metabolic pathways

Research on the interaction between the gut microbiome and immunity is an emerging field that examines the role of environmental factors, such as diet, as well as genetic and immune signals in metabolism, immunity, and host response to infection [58]. Studies on immune dysregulation may contribute to our understanding of the effects exerted by the microbiome in cancer development and treatment. In patients with colorectal cancer (CRC), the gut microbiome can directly or indirectly affect CRC by secreting metabolites, invading tissues, and modulating host immune response [39, 40]. Clostridium, Peptostreptococcus, Porphyromonas Genus, Prutella, Bacteroides, and twin cocci are the most significant bacteria associated with CRC [41, 59].

The liver is sensitive to intestinal bacterial metabolites, and changes in the intestinal microbiome affect the function of immune cells in the liver. Moreover, commensal microbiota can mediate the metabolism of primary to secondary bile acids. Gut microbiota are thought to be involved in the physiological activities of the host by affecting the bile acid pool, thus regulating hormone secretion and immunity via the resulting metabolites [60, 61]. We hypothesize that the gut microbiome promotes host immunity mainly through anabolic pathways. Ma et al. have found that the immune response to liver cancer has the opposite effect and that reducing the abundance of intestinal Clostridial bacteria through the use of antibiotics can increase the levels of primary bile acids and inhibit liver tumors by increasing the expression of CXC chemokine ligand 16 (CXCL16) in sinusoidal endothelial cells, after primary bile acids are metabolized to secondary bile acids by Clostridium bacteria [27]. Primary bile acids increase CXCL16 expression, whereas secondary bile acids exert the opposite effect. Schramm et al. have reported that the expression of CXCL16 in patients with liver cancer is linked to primary bile acids. However, there are differences in the composition of human and mouse immune systems, intestinal microbiome, and bile acid [62]. Therefore, the clinical significance of the relevant research results is limited.

**Fig. 2:**
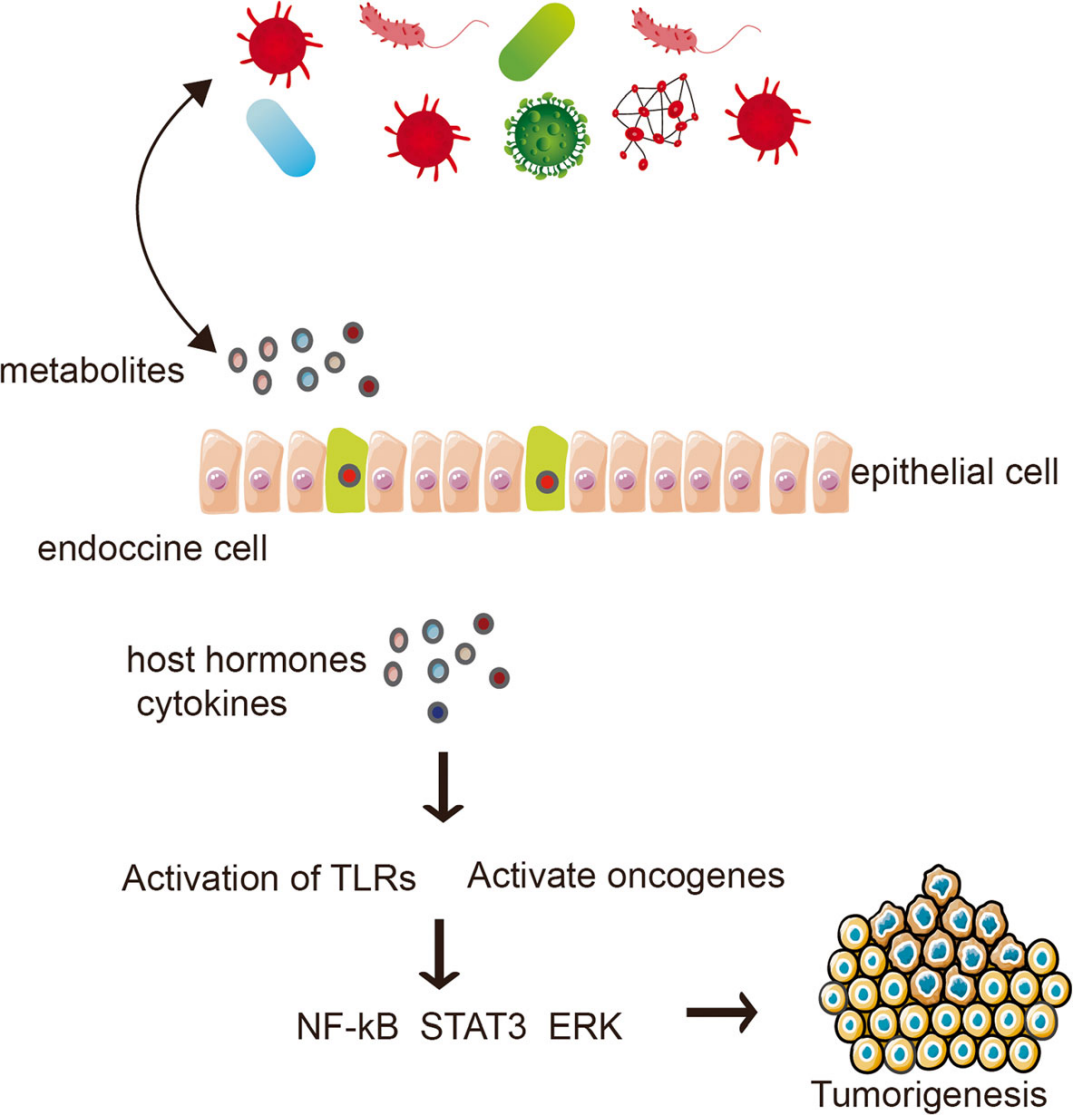
Microbiota metabolites are related to tumor development. Pro-inflammatory pathways are activated when the mucosal barrier is broken. The loss of the boundary between the host and the microorganism is related to the pattern recognition receptor signaling cascade. The feedforward circuit of chronic inflammation mediated by NF-kB and STAT3 signal transduction promotes canceration in transformed cells and non-tumor cells. Microbial PAMP activate TLR signaling in a variety of cell types, leading to cytokine production and NF-kB-mediated inflammation, thereby exacerbating tumor growth

In mice, gut microbiota and bile acid products play diverse roles in cancer development. For example, elimination of Clostridium XIV, increase in primary bile acids, and reduction in secondary bile acids inhibits the progress of liver cancer. Immune cells, such as dendritic cells, macrophages, and myeloid-derived suppressor cells, can be regulated by bile acids or their receptors, thus promoting anti-cancer immune responses. Bile acid receptors and flora metabolites may be novel targets for cancer treatment in the future [63]. Moreover, microbial pathogen-associated molecular patterns can activate toll-like receptor (TLR) signaling in a variety of cell types, leading to cytokine production and NF-κB-mediated inflammation, which can fuel tumor growth. Gut microbiota exacerbate metabolic inflammation through TLR signaling [64]. For example, the LPS receptor TLR4 has been shown to promote hepatocellular carcinoma, pancreatic cancer, and colon cancer. TLR-induced activation of NF-kB and STAT3 is a key signaling pathway that promotes cancer [32]. NF-κB signaling can stimulate glycolytic energy flux during acute inflammation [65]. (Fig. 2).

### Gut microbiota may affect the efficacy of PD-1 inhibitors

Previous studies in mouse models and humans have shown that regulation of the fecal microbiome significantly affects the outcome of cancer immunotherapy in terms of toxicity and efficacy [66]. Cancer immunotherapy based on the blockade of programmed cell death protein 1 (PD-1) and programmed death-ligand 1 (PD-L1) has become an essential approach for the treatment of various cancers in advanced stages [66]. Fecal bacterial transplantation may alter the gut microbiome of patients with cancer to improve the efficacy of drugs such as anti-PD-1 monoclonal antibody. Routy et al. have found that the response of patients with lung or kidney cancer to PD-1 monoclonal antibody is related to a higher abundance of Akkermansia muciniphila [67].

It is worth noting that the efficacy of tumor immunotherapy is related to the composition of intestinal bacteria [4]. Gopalakrishnan et al. have shown that, in patients with melanoma, the response to treatment with PD-1 monoclonal antibody was related to Faecalibacterium-based flora in patients [68]. There is a link between the efficacy of the cancer immune drug PD-1 blocker and the gut microbiota of patients, as gut microbiota may affect the efficacy of PD-1 inhibitors. Scientists suspect that the cytokine IL-12, which is released in response to A muciniphila, may help recruit T cells to combat cancer [3, 69]. Cancer patients harboring diverse intestinal flora rich in Clostridium lentum and Clostridiaceae have a better response to PD-1 inhibitors, and may show more significant effects of immunotherapy [3, 4].

A study examining the response of patients with different flora to PD-1 monoclonal antibodies has shown that responding patients were found to have a higher diversity of bacteria and a higher abundance of Bifidobacterium longum [69]. Therefore, the composition of the gut microbiome can affect the anti-tumor immunity of cancer patients, and can be used as a biomarker to predict the response of patients to immune checkpoint blockade therapy.

Prediction of the efficacy of PD-1 through microbial signals may require combining RNA sequencing, metabolomics, cancer process management, and intestinal fungal/viral analysis through clinical trials to fully understand the relationship between microbiota and the efficacy of tumor immunotherapy [70, 71]. Gut microbiota can regulate anti-PD-1 efficacy by interacting with the host immune system and thus represent a new therapeutic target [68, 72].

### Patients with cancer should use antibiotics judiciously

Cancer treatment with microbial preparations or their products has the potential to shrink tumors [73]. Because flora can affect cancer progression, it may also affect the efficacy of chemotherapy and immunotherapy. Intervention with fecal flora affects the toxicity and effectiveness of immunotherapy. Gut microbes have been considered as key modulators of host immunity, raising the possibility that they could influence the outcome of cancer immunotherapy [74]. For example, it has been suggested that the use of antibiotics early in the treatment reduces the survival rate of patients with renal cell carcinoma and non-small cell lung cancer [66]. Antibiotics have saved countless lives, but they have also many side effects, such as *Clostridium difficile* infection, antibiotic resistance, and flora changes [75]. A study by Wu et al. has found that the use of antibiotics increases the risk of colon cancer, but reduces the risk of rectal cancer [76]. Antibiotic exposure is the main reason for the emergence of drug resistance, leading to the accumulation of drug-resistant genes in specific locations; the abundance and diversity of drug-resistant genes in the intestinal flora are high, which may be closely related to the use of antibiotics [77].

## Gut microbiota dysbiosis affects cancer through gut-organ axis

The gut-organ axis establishes links or a two-way or multi-directional communication between organs through nerve, endocrine, immune, humoral, and metabolic pathways [78]. Intestinal flora and their secretions can be involved in the occurrence and development of tumors through the gut-organ axis [79, 80]. The bidirectional relationship between the gut and vital human organs (such as the lung, brain, and liver) is discussed below.

### Gut microbiota dysbiosis affects cancer through the gut-brain axis

The gut-brain axis plays an important role in tumor proliferation, invasion, apoptosis, autophagy, and metastasis [47, 81]. Ruty et al. have proposed that gut microbiota can follow many routes to the brain and impact brain tumor therapeutic interventions [82]. In support, gut microbiota has been shown to have significant associations with cancer treatment-related psychoneurological symptoms [83].

The gut microbiota-brain axis includes gut microbiota and their metabolic products, the enteric nervous system (ENS), sympathetic and parasympathetic branches within the autonomic nervous system, neural-immune system, neuroendocrine system, and central nervous system (CNS) [84]. The gut microbiome produces most neurotransmitters found in the human brain [85, 86]. Recent studies have shown that the CNS and ENS can interact with gut microbiota to regulate nutrient metabolism. The vagal nerve system facilitates communication between the CNS and ENS to control gastrointestinal tract functions and feeding behavior. Vagal afferent neurons also express receptors for gut peptides that are secreted from enteroendocrine cells such as cholecystokinin (CCK), ghrelin, leptin, peptide tyrosine tyrosine (PYY), glucagon-like peptide-1 (GLP-1), and 5-hydroxytryptamine (5-HT; serotonin). The gut microbiome can regulate the levels of these peptides to influence the vagal afferent pathway and thus regulate intestinal metabolism via the microbiota-gut-brain axis. Serotonin functions as a key neurotransmitter at both terminals of this network. Local alterations in serotonin concentrations with subsequent relay of signals along the brain-gut axis influence CNS neurotransmission and regulate the function of neural processes in the gastrointestinal tract [87]. Gut microbiota dysbiosis can result in changes in serotonin levels. In triple-negative breast cancer, it has been shown that serotonin promotes cancer progression through autocrine serotonin signaling [48, 49, 88]. Dysbacteriosis-mediated expression of the glucagon-like peptide-1 has been shown to affect autophagy in endometrial cancer and is related to the occurrence of pancreatic cancer [89, 90].

### Gut microbiota dysbiosis affects cancer through the gut-liver axis

**Fig. 3:**
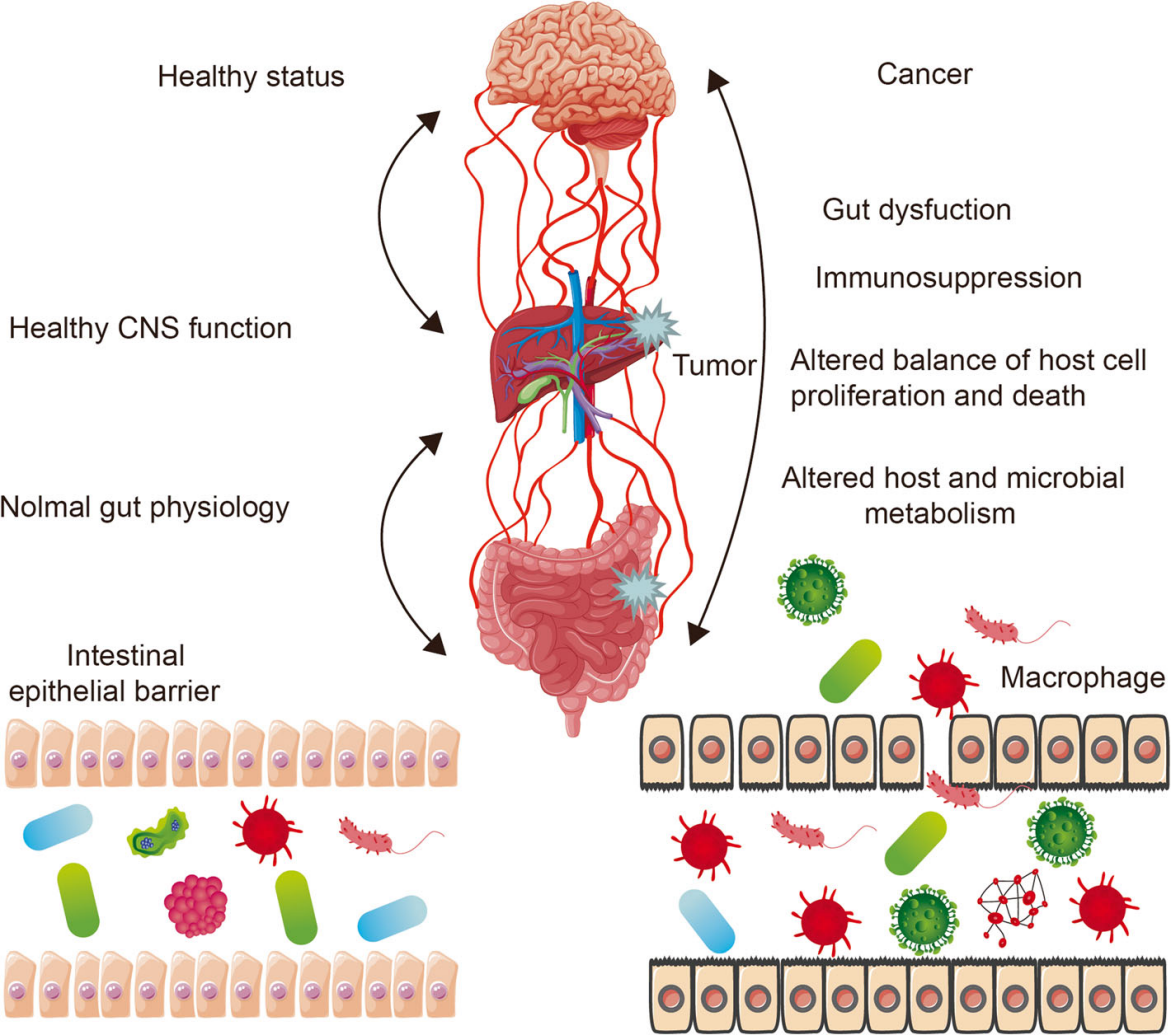
The dysbacteriosis leads to continuous destruction of the barrier and delayed restoration of homeostasis. In these cases, the microbiota can affect canceration by changing the proliferation and apoptosis of host cells, disrupting the function of the immune system, and affecting the metabolism of the host

The gut microbiome may contribute to cancer pathogenesis and progression through the gut-liver axis [91]. The two-way relationship between the intestine, microbiota, and liver integrates signals generated by dietary, genetic, and environmental factors [92]. This reciprocal interaction is facilitated by the portal vein [93]. Dysbiosis of gut microbiota leads to the progression of chronic inflammation and liver disease, thereby increasing the risk of HCC [35, 42]. Of note, bile acid and LPS provide an important link between the liver, bacterial microbiota, and the intestine. Intestinal microbiome-mediated bile acid metabolism regulates liver cancer through natural killer cells [27]. In a mouse model with dysbiotic intestinal flora, the synthesis of long-chain fatty acids is reduced, accompanied by oxidative stress and inflammation [94]. Bile acid, short-chain fatty acids (SCFAs), trimethylamine-N-oxide (TMAO), and immunoglobulin A (IgA) can also exert metabolic control through the microbiota-gut-liver axis. Further research should focus on the role of gut microbiota in the neuroendocrine regulation of nutrient metabolism via the microbiota-gut-brain-liver axis [95–97]. Intestinal microbes can promote cancer progression by changing the balance between host cell proliferation and death, and by affecting the immune system (Fig. 3).

### Gut microbiota dysbiosis affects cancer through the gut-lung axis

The human respiratory tract is the main and continuous entrance for many microorganisms and particles (such as viruses and bacteria), and the lung is an environment rich in flora [98, 99]. The intestinal flora may also have an impact on lung health. Changes in the microbial composition and function of the intestine are related to the development of lung diseases [100]. Metabolites such as SCFAs are produced by the microbiota and may regulate inflammation in the lungs [101, 102]. It appears that chronic lung diseases, such as cancer, are linked to dysbiotic airway microbiota and commonly occur alongside GI disorders [103, 104].

There is increasing evidence of a close relationship between the gastrointestinal tract and the respiratory tract. The exacerbation of chronic intestinal and lung diseases has key conceptual features related to the disorder and imbalance of the microbial ecosystem [105]. The surviving bacteria, cell wall fragments, or protein fragments of dead bacteria escape along with the cytokines and chemokines produced in the intestine, and then enter the general circulation. Entering the pulmonary circulation may lead to the activation of dendritic cells and macrophages and differentiation of T cells [106]. The concentration of circulating SCFAs in the intestine affects IL-6 and IL-8 in lung cancer and is related to the occurrence and development of lung cancer [107, 108]. In addition, patients with non-small-cell lung cancer experience gut butyrate-producing bacterial dysbiosis [109]. A significant relationship has also been found between *Mycobacterium tuberculosis* (TB) and lung cancer [43, 44]. A possible reason is that persistent tuberculosis infection can cause the production of tumor necrosis factor and cause lung inflammation. In addition, pulmonary fibrosis caused by TB leads to the synthesis of extracellular matrix, which is involved in the development of lung cancer [110, 111].

## Conclusion and perspectives

Dysregulation of the gut microbiota and its interaction with the host may be important in tumorigenesis. First, we need to identify relevant bacteria in humans, study their abundance and the impact of their products on cancer progression, and elucidate their interactions with the human immune system as well as their ultimate impact on the mechanism of tumor occurrence and development. We then need to identify novel therapeutic microbial interventions and combine them with conventional therapies to treat tumors and other multifactorial human diseases.

## Data Availability

Not applicable.

## Abbreviations

PD-1: Programmed cell death protein 1
PD-L1: Programmed death-ligand 1
NOD2: Nucleotide-binding oligomerization domain 2
HPV: Human papillomavirus
SYDS: Pleen-yang-deficiency syndrome
CCL5: C-C chemokine ligand 5
CXCL16: CXC chemokine ligand 16
PAMPs: Pathogen-associated molecular patterns
IL-6: Interleukin-6
IL-12: Interleukin-12
CNS: Central nervous system
ENS: Enteric nervous system
EECs: Enteroendocrine cells
CCK: Cholecystokinin
PYY: Peptide tyrosine tyrosine
GLP-1: Glucagon-like peptide-1
5-HT: 5-hydroxytryptamine
HCC: Hepatocellular carcinoma
SCFAs: Short-chain fatty acids
TMAO: Trimethylamine-N-oxide
IgA: Immunoglobulin A
SCC: Small cell carcinoma
AC: Adenocarcinoma
NSCLC: Non-small-cell lung cancer
TB: Tuberculosis
TNF: Tumor necrosis factor
ECM: Extracellular matrix
SARS-Cov-2: Severe acute respiratory syndrome coronavirus-2
COVID-19: Coronavirus disease 2019
ACE2: Angiotensin-converting enzyme 2
LPS: Lipopolysaccharide
