# Correction to: Gut microbiota influence tumor development and Alter interactions with the human immune system

**DOI:** 10.1186/s13046-021-02131-1

**Published:** 2021-10-25

**Authors:** Yanshan Ge, Xinhui Wang, Yali Guo, Junting Yan, Aliya Abuduwaili, Kasimujiang Aximujiang, Jie Yan, Minghua Wu

**Affiliations:** 1 Hunan Cancer Hospital and the Affiliated Cancer Hospital of Xiangya School of Medicine, Central South University, Changsha, 410013 Hunan China; 2 Basic School of Medicine, Central South University, Changsha, 410078 Hunan China; 3 The Key Laboratory of Carcinogenesis of the Chinese Ministry of Health, The Key Laboratory of Carcinogenesis and Cancer Invasion of the Chinese Ministry of Education, Cancer Research Institute, Central South University, Changsha, 410008 Hunan China; 4 Basic School of Medicine, Xinjiang Medical University, Urumqi, 830011 Xinjiang China; 5 Department of Forensic Science, School of Basic Medical Sciences, Central South University, Changsha, 410078 Hunan China

**Correction to: J Exp Clin Cancer Res 40, 42 (2021)**

**https://doi.org/10.1186/s13046-021-01845-6**

Following publication of the original article [1], author has corrected affiliation 1 details. The correct affiliation should be:

^1^Hunan Cancer Hospital and the Affiliated Cancer Hospital of Xiangya School of Medicine, Central South University, Changsha 410013, Hunan, China.

The correction does not have any effect on the results or conclusions of the paper. The original article has been corrected.
